# Multiplexing AAV Serotype-Specific Neutralizing Antibodies in Preclinical Animal Models and Humans

**DOI:** 10.3390/biomedicines11020523

**Published:** 2023-02-11

**Authors:** Hisae Kuoch, Karina Krotova, Melanie L. Graham, Mark L. Brantly, George Aslanidi

**Affiliations:** 1 The Hormel Institute, University of Minnesota, 801 16th Avenue NE, Austin, MN 55912, USA; 2 Department of Surgery, Medical School, University of Minnesota, Minneapolis, MN 55108, USA; 3 Division of Pulmonary, Critical Care and Sleep Medicine, Department of Medicine, Medical School, University of Florida, Gainesville, FL 32610, USA

**Keywords:** adeno-associated virus, serotype-specific neutralizing antibodies, assay development, patient population, preclinical animal model

## Abstract

The accurate assessment of AAV-specific pre-existing humoral immunity due to natural viral infection is critical for the efficient use of clinical gene therapy. The method described in the present study applies equivalent infection conditions to each AAV serotype (AAV1, AAV2, AAV3, AAV5, AAV6, AAV7, AAV8, AAV9, AAV10, and AAVAnc80L65). In the current study, we validated the assay by assessing AAV-neutralizing antibody titers in a limited cohort of random human donors and well-established preclinical large animal models, including dogs and non-human primates (NHPs). We achieved a rapid and accurate evaluation of neutralizing titers for each individual subject that can be used for clinical enrollment based on specific AAV serotypes and individualized selection of the most suitable AAV serotype for each specific patient.

## 1. Introduction

AAV-based treatments for monogenic diseases have experienced major success, as two therapies recently became available for clinical practice [1,2]. In recent years, possibilities for AAV-based treatment have continued to expand towards applications in cancer [3] and infectious diseases [4]. The safety profile of such treatments is among the most attractive features of recombinant AAV vectors since wild-type AAV (wt) is widely accepted as an unequivocally harmless virus. However, regardless of the non-pathogenic nature of the AAV, genuine infection is found to be common among humans and can be wide-ranging depending on the AAV serotype, appearing in 30–80% of all tested individuals. Infection-induced serotype-specific neutralizing antibodies (NABs) may result in the exclusion of a large patient population from novel therapeutic options such as AAV-based gene therapy [5,6,7,8,9,10,11,12,13,14]. The same concern applies to the two most recognizable animal models widely used for preclinical evaluation of the efficacy and safety of AAV-based gene therapies, non-human primates (NHPs) and dogs [15,16,17,18,19,20].

The seroprevalence against wild-type (wt) AAVs in humans varies dramatically depending on the method used for analysis, the origin and composition of the AAV capsid, and especially the geographic location of the tested population. There is a lack of systematic studies, however, that address seroprevalence to different AAV serotypes in the general population. The data are usually collected as needed for particular clinical trials and usually focus on a single AAV serotype. The prevalence of AAV serotypes that are commonly used in clinical applications can be as low as 30% for AAV5 or 40% for AAV8 and as high as 80% for AAV1 and AAV2 serotypes, according to some studies in the US and the EU [7,8,9,10,11,12,21,22,23,24]. A study in the UK identified the seroprevalence of NABs for AAV3B, its recombinant library-derived variation AAV-LK03, and AAV8, which reached up to 23%, 35%, and 18% prevalence, respectively, [25] and up to 30% for AAV6 vectors [26]. An analogous study in India revealed a prevalence of NABs for AAV3 of up to 80–90% in both healthy individuals and hemophilia B patients [27]. An additional challenge for the use of a specific AAV vector for clinical gene therapy is the substantial cross-reactivity among evolutionarily similar AAVs, which can reach 50–60% depending on the tested AAV serotypes [21,25]. 

Unlike the cellular immune responses to AAV capsids, which can be avoided with transient immunosuppression using corticosteroids or other immunosuppressive drugs [28], humoral immunity is more challenging to mitigate. One way to avoid humoral responses is to treat newborns with AAV-based gene therapy before they develop NABs against AAV [25,29]; however, this approach cannot always be applied because genetic screening is not widely available, and, in many cases, the genetic disease is not identified until physiological outcomes become obvious. 

To circumvent problems associated with pre-existing immunity, many novel and modified AAV variants, which can avoid neutralization by the host, were developed through rational design based on reconstruction of the capsid structure [30,31], pulled from recombinant libraries [32,33,34,35,36,37,38,39], or isolated from different species, such as swine, caprine, and snakes. As a rule, these new variants are not cross-reactive with commonly used AAV serotypes derived from humans or NHPs [40,41,42], and their therapeutic applications are currently being studied in preclinical models and early-stage clinical trials to determine their effectiveness and safety. 

At the same time, the pharmacological reduction of AAV NABs was explored using IgG-degrading enzymes and IgG-cleaving endopeptidase, which were able to deprive AAV-specific IgG in dog, monkey, and human antisera and allow AAV administration in mice and NHP models regardless of the presence of NABs [43,44]. Additionally, the immune adsorption of NABs has yielded some positive results for gene delivery via AAV5 and AAVrh.74 in NHPs [45,46] and AAV1, 2, and 6 in humans [47]. However, the ability to overcome pre-existing immunity in these studies was tested on relatively modest NAB titers and might not be useful for patients with high neutralizing capacities. 

Another approach to overwhelming NAB capacity is the administration of high doses of AAV capsids or a mixture with an empty decoy capsid [48]; however, this protocol maximizes the risk of activating the anti-capsid T-cell response and might lead to inactivation of therapeutic gene expression over time.

Regardless of the efforts described above to overcome the negative effects of AAV NAB, the accurate measurement of titers is absolutely critical for the proper enrollment of patients in clinical studies and adequate comparison between multiple clinical trials. Currently used assays for evaluating AAV seroprevalence lack standardization through the spectrum of AAV serotypes used in clinical research. The present study was performed to evaluate the pertinence of our recently developed AAV serotype universal assay [49] to assess the titers of AAV NABs against multiple commonly used AAV serotypes in blood samples in a limited cohort of human subjects, dogs, and NHPs. We confirmed the ability of our new NAA to rapidly and simultaneously evaluate the inhibitory properties of individual blood samples with a wide range of NAA titers against ten AAV serotypes. As a result of this assessment, the seroprevalence for certain AAV serotypes and the lack of specific NABs against other serotypes can be determined for each individual patient. On the preclinical side, animals can be properly selected to accurately model the intended clinical cohort and enhance predictions of treatment outcomes. In clinical studies, our method optimizes the selection of an ideal cohort of patients for therapeutic gene therapy and, at the same time, provides an opportunity for a personalized approach by identifying the most appropriate AAV serotype for each specific patient. 

## 2. Materials and Methods

### 2.1. Human Samples

De-identified human plasma samples were provided by the University of Florida. Sample collection was performed as part of the study “The role of conformational diseases on macrophage function” and approved by the Institutional Review Boards at the University of Florida (IRB #2011501051). 

### 2.2. Animal Samples

Plasma samples from individual dogs, both males and females, were obtained from two independent commercial vendors (Innovative Research, Minneapolis, MN, USA and BioChemed, Winchester, VA, USA). 

NHP samples were provided by the University of Minnesota. All procedures in NHPs were approved by the University of Minnesota Institutional Animal Care and Use Committee (1903-36845A), were conducted in compliance with the Animal Welfare Act, and adhered to the principles stated in the *Guide for Care and Use of Laboratory Animals.* A total of 6 healthy cynomolgus macaques (*Macaca fascicularis*) (female = 3, male = 3) were used. All animals were purpose-bred and purchased from institutionally approved commercial vendors. To facilitate cooperative blood sample collection, animals were trained using a positive reinforcement paradigm [50]. Blood samples were collected from awake, cooperating animals via a subcutaneous vascular access port [51]. At the time of sampling, NHPs were aged between 3.0 and 7.3 years (median 3.9 years) and weighed between 2.9 and 7.0 kg (median 3.7 kg).

### 2.3. Plasma Samples Preparation

All samples were collected with non-heparin tubes to avoid non-assay-related inhibition for certain AAV serotypes, such as 2 and 6. FBS (Gibco, ThermoFisher, Waltham, Massachusetts, USA) was used for diluting tested serum samples. All serum was heat-inactivated at 56 °C for 30 min before use.

### 2.4. AAV Vector Production and Purification

The single-stranded AAV vectors (serotypes 1, 2, 3, 5, 6, 7, 8, 9, 10, and Anc80L65 [52]) expressing the firefly luciferase gene [49] were packaged in adherent HEK293 cells via triple transfection with PEI (Polyscince, Warrington, PA, USA) and purified using an iodixanol (Millipore-Sigma, Billerica, MA, USA) density gradient followed by ion-exchange column (Cytiva, Piscataway, NJ, USA) chromatography, as described before [53,54]. Concentrated AAV stock titers were determined on DNase I (ThermoFisher, Waltham, MA, USA)-resistant particles via qPCR with primers specific for the CMV enhancer of the chicken-β-actin promoter [53,54]. 

### 2.5. AAV-Neutralizing Antibody Assay

**Figure 1:**
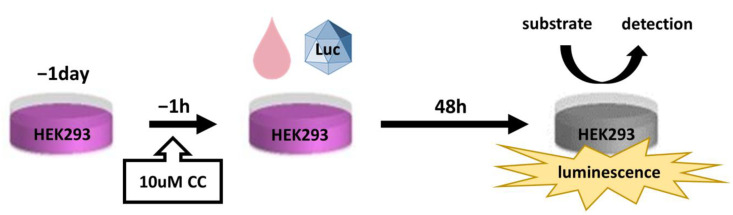
Schematic outline of the experimental procedure neutralizing antibody assay.

The detailed protocol has been described previously [49]. Briefly, AAV vectors of ten different serotypes expressing luciferase as a reporter gene were incubated with serial dilutions of a tested serum sample and then added to HEK293 cells pre-treated with 10 μM of Compound C (CC) (Millipore-Sigma, Billerica, MA, USA) at the same MOI of 2000 vg/cell. The infection efficiency was estimated 48 h later via luciferase activity in infected cells measured using the chemiluminescence produced by the degradation of the Bright-Glo (Promega, Madison, WI, USA) substrate. AAV NAB titers were determined as the dilution of the tested serum sample at which 50% of the chemiluminescence read off was inhibited compared to the maximum signal set at 100%, determined based on the control (AAV in the presence of the diluent only). Nonlinear regression fit was performed using Graph Pad Prism 7 software (GraphPad Software, Boston, MA, USA) to obtain the NAB titer from the saturation curve. Cells not infected with AAV were used to establish the baseline fluorescent signal). The schematic design is illustrated in Figure 1.

### 2.6. Statistical Analysis

*All data* are shown as the mean ± SD. For all statistical analyses, an unpaired t-test was used to compare the chemiluminescence signals from corresponding AAV pre-incubated with tested serum with the control of AAV in the presence of the diluent only. Data were considered significant when *p* values were <0.05.

## 3. Results

### 3.1. Evaluation of AAV NABs in Human Samples

**Figure 2:**
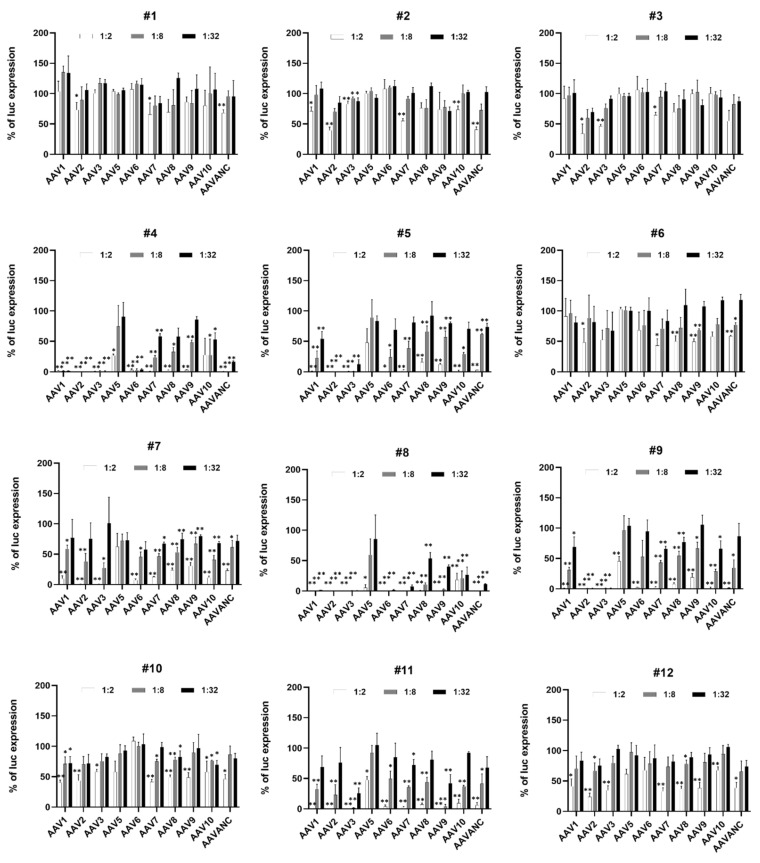
Screening of human samples for the presence of anti-AAV NAB. Human serum samples (*n* = 12) were screened for the presence of NAB against 10 AAV serotypes. For the initial screening, tested serum samples were used at dilutions 1:2, 1:8, and 1:32, and FBS was used as a diluent. The data are presented as a percentage of luciferase activity in the presence of the corresponding dilution of tested samples compared to the control (AAV serotype X- Luc mixed with diluent only). Each column is a mean ± SD in triplicate. Statistically significant differences are indicated as *p* * < 0.05, *p* ** < 0.01 vs. the uninhibited control for each AAV serotype.

**Figure 3:**
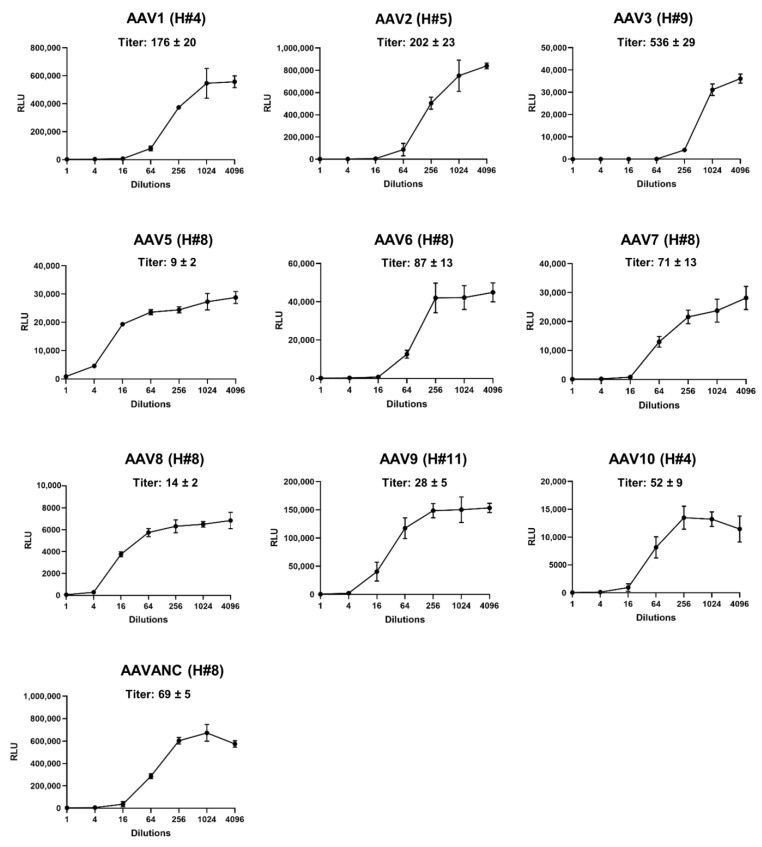
The determination of anti-AAV NAB titers in selected human serum samples. Human samples with titers higher than 1:32 against particular AAV serotypes were re-evaluated at dilutions ranging from 1:2 to 1:4096, and inhibitory curves were built to determine the titers. Since the NAB titers for AAV5 and AAV8 in all tested human samples were lower than 1:32, an assay was performed for several random samples as a proof of concept. The Y-axis presents chemiluminescence intensity units of the reporter luciferase encoded by AAV. NAB titers were determined as a dilution of the serum sample at which 50% of the fluorescence signal was obstructed. The representative inhibitory curve for each analyzed AAV serotype is shown.

The observations from several human clinical trials suggested that the presence of even relatively low NAB titers (≥1:5), which likely occur from natural AAV exposure, can inhibit AAV-mediated therapeutic gene activity, and limit the efficacy of gene therapy [13,29]. Thus, an accurate, serotype-independent, and sensitive evaluation of AAV-specific NABs provides key information that defines the eligibility of patients for AAV-vector-based gene therapy. To demonstrate that the presence of NABs against different AAV serotypes can be rapidly and uniformly estimated, we performed screening of serum samples collected from random human volunteers. To perform a well-timed analysis with maximum efficiency, the next strategy was applied. In the initial screening, three dilutions (1:2, 1:8, and 1:32) of each blood sample were used to build the inhibitory profile of luciferase activity mediated by different AAV serotypes. The initial analysis with limited dilutions allowed quick estimation of NAB levels and separated the serum samples without NABs and/or with low titers (≤2) against particular AAV serotypes from the samples with a high inhibitory capacity (Figure 2). For seven out of twelve human samples (# 1–3, 6, 7, 10, 12), the NAB titers were lower than 1:32 for all ten serotypes; hence, these samples did not require additional analysis. The rest of the samples (#4, 5, 8, 9, 11) had NAB titers higher than 1:32 against at least one of the AAV serotypes. For these samples, the assay was repeated at dilutions from 1:2 to 1:4096 (Figure 3). This range of dilutions was sufficient to determine the titers for all tested human samples except for AAV2 in sample #5, for AAV7 in sample #8, and for AAV3 in sample #9, which did not reach a plateau at the highest dilution of 1:4096. NABs with titers ˃ 1:2 against AAV 2 were detected in 11 out of 12 human samples, making this the most prevalent serotype in the tested samples and confirming that humans are natural hosts for AAV2 [55]. The NAB against AAV1 and AAV3 was the next most frequent antibody in human samples. All tested subjects showed an absent-to-low prevalence of AAV5. Our data are in agreement with several independent evaluations of NABs against AAV5, which is considered to have the lowest rate of pre-existing NABs, with as low as approximately 20–30% of tested humans being seropositive, and also has the potential to target a larger number of patients in the development of AAV5-based gene therapy [10,23]. A summary of the overall results for each subject is provided in Table 1. A color code is used for better visual presentation of the data: blue, yellow, brown, and red correspond to AAV NAB titers ≤2, 2–8, 8–32, and ≥32, respectively. 

### 3.2. Evaluation of AAV NABs in Large Animal Models

**Figure 4:**
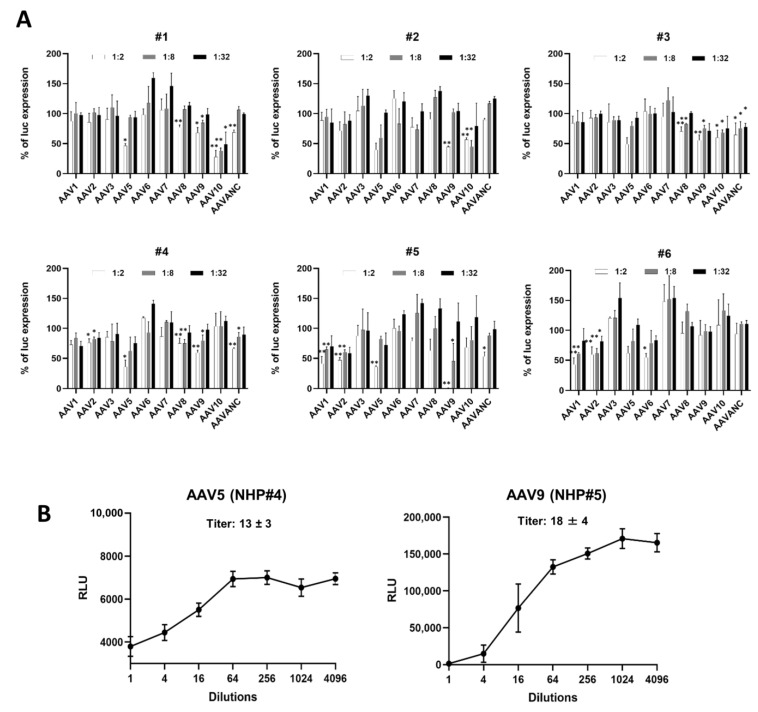
Anti-AAV NAB assay for non-human primate (NHP) samples. (**A**) Screening for the presence of NABs against 10 AAV serotypes in NHP serum (*n* = 6). Samples were screened as described for human samples in Figure 2 at dilutions of 1:2, 1:8, and 1:32. Data are presented as the mean ± SD in triplicate. Statistically significant differences are indicated as *p* * < 0.05, *p* ** < 0.01 vs. uninhibited control for each AAV serotype. (**B**) NAB titers for AAV5 in NHP sample #4 and AAV9 in NHP sample #5 determined at dilutions ranging from 1:2 to 1:4096.

**Figure 5:**
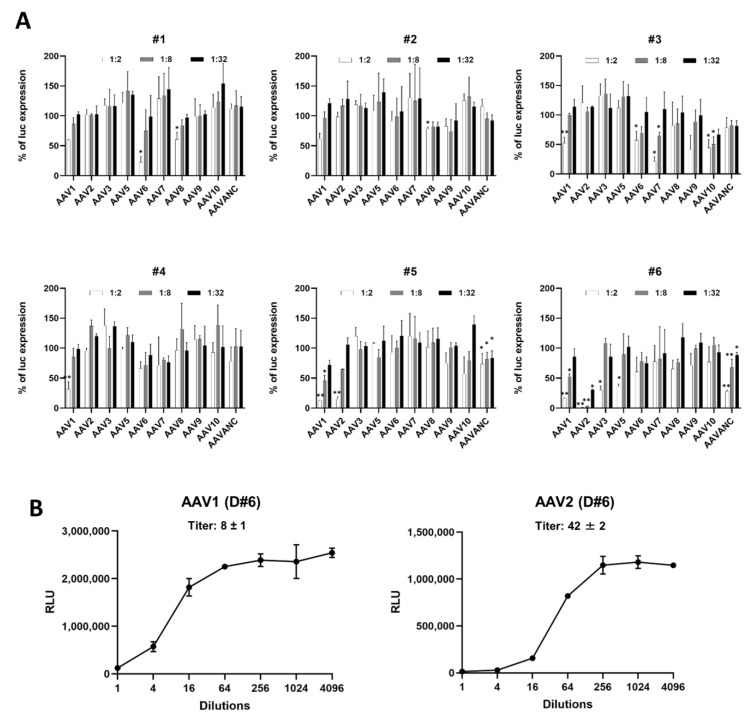
Anti-AAV NAB assay for dog samples. (**A**) Screening for the presence of NABs against 10 AAV serotypes in dog serum samples (*n* = 6). Samples were screened as described above in Figure 2 and Figure 4 at dilutions of 1:2, 1:8, and 1:32. Data are presented as the mean ± SD in triplicate. Statistically significant differences are indicated as *p* * < 0.05, *p* ** < 0.01 vs. the control. (**B**) NAB titer for AAV1 and AAV2 in dog sample #6 determined at dilutions ranging from 1:2 to 1:4096.

The use of animal models other than mice models is vital for the successful evaluation of novel AAV-based gene therapies prior to human clinical trials, as such models increase the accuracy in efficacy evaluations of vector performance and potential side effects. Dogs and NHPs, similarly to humans, are naturally exposed to AAV and, therefore, closely recapitulate the inhibitory effects of pre-existing humoral immunity against AAV capsids, as well as the outcomes of gene therapy treatment. Dog models have been used extensively to evaluate the efficacy of AAV-based gene therapy for treatments of hemophilia, muscular dystrophies, and retinal genetic abnormalities prior to human use [56,57,58,59,60,61,62]. NHPs are the closest genetic relatives to humans, exhibiting similarities in major organ anatomy, cell physiology, and immunology [63,64,65,66,67,68,69]. NHPs have been successfully used as a disease-free preclinical model for AAV-based therapy [70,71,72,73]. Thus, in this study, we utilized blood samples from six dogs and six NHPs to evaluate their inhibitory properties against ten AAV serotypes and the prevalence of serotype-specific NABs. The same two-way strategy used for screening human samples with a limited number of dilutions and the construction of a saturation curve was applied to analyze the levels of NAB in animal samples. The six analyzed NHP samples had low or moderate titers (≤32) for all 10 AAV serotypes (Figure 4A). To identify the exact titer, the assay was repeated with a wide spectrum of dilutions for several random samples (Figure 4B). For AAV serotypes 3, 6, 7, 8, and ANC, the titers were less than or equal to 1:2 in all analyzed samples. Specific NABs for serotypes 1, 2, 9, and 10 were detected in one or two samples out of six. NABs against AAV5 were most prevalent in this particular cohort of NHPs and detected in four out of six samples. In six dog samples analyzed in this study, all titers were moderate. Only in sample #6 was the titer for NABs against AAV2 higher than 1:32 (Figure 5A,B). For AAV1, three out of six dog samples had titers between 1:8 and 1:32, and no dogs were identified as being positive for AAV8. NABs against other serotypes were only detected in one or two samples. No animals were completely free of AAV NABs, and each individual dog was positive for at least one of each tested AAV serotype. Surprisingly, none of the dogs tested positive to AAV6 serotypes described as being the most seroprevalent in prior studies [59,74,75], which might be attributable to the particular geographical environments in which the blood samples were collected. A summary of the overall results for each dog and NHP sample is provided in Table 2. Similar to the human summary table, a color code is used for better visual presentation of the data: blue, yellow, brown, and red correspond to AAV NAB titers ≤2, 2–8, 8–32, and ≥32, respectively. 

## 4. Discussion

AAV-based therapy is a popular platform for clinical gene delivery with a growing number of targeted diseases. In 2019, 288 AAV-based clinical trials were registered with the US FDA database. Of these, 149 were identified as unique clinical trials [76]. However, widespread infection by wt AAV and the resulting presence of circulating NABs in a large proportion of the human population represent a major hurdle for the successful clinical application of AAV therapy. Since the presence of NABs even at low titers can affect gene therapy efficiency, NAB titers are a major exclusion criterion for AAV-based clinical studies [77]. 

Furthermore, the threshold for NAB titers significantly varies from 1:1 to 1:80, depending on the design of clinical studies, particularly the route of vector administration [21,78,79,80]. Clinical trials based on intramuscular delivery have the least strict exclusion criteria and can allow NAB titers up to 1:50 and, in rare cases, even higher. In the clinical trial for alpha-1-antitrypsin (AAT) deficiency, intramuscular gene delivery produced similar AAT levels in two subjects with pre-existing NAB titers of 1:80 and 1:160 against AAV1 vectors; these results are similar to those of individuals testing as seronegative [48]. At the same time, pre-existing immunity has a profound effect on liver-directed AAV gene therapy, and NAB titers as low as 1:5 completely block hepatocyte transduction, which leads to far more rigorous criteria for patient enrollment compared to muscle-directed gene transfer [14,78]. 

It is important to note that the methods used to define AAV serotype-specific NABs vary significantly between clinical trials, which leads to inconsistencies in the definition of seropositivity for different populations of patients. 

Currently, several methodologically different assays are used for the preclinical and clinical determination of AAV NAB titers and neutralizing activities, including ELISA [79], a qPCR-based AAV-to-cell binding assay [80], and the in vitro inhibition of AAV activity through the presence of serial dilutions of a tested sample [81,82,83] or similar in vivo assays with serum or IgG in known concentrations injected in mice [84,85]. However, some of these methods, such as those using in vivo NAB activity, are lengthy and cost-ineffective, and some measure the total AAV-specific antibodies without analyzing their neutralization properties. As an example, some of the clinical trials estimated total antibody titers against AAV capsids using ELISA assays, which do not necessarily adequately address the neutralizing capacities of the samples. Indeed, some AAV-binding antibodies do not have neutralizing activities and instead promote AAV-based gene transfer [86]. Hence, ELISA-based data might lead to the unnecessary exclusion of prospective patients who would benefit from this therapy. 

In contrast, in vitro assays based on the inhibition of AAV-expressed reporter gene activity are easy to set up and reproduce, and, importantly, these assays are recommended by the US Food and Drug Administration (FDA) for patient screening and AAV-treatment-mediated NAB evaluations in clinical trials [87]. However, difficulties remain in pinpointing a single cell line that is equally permissive for infection with a variety of AAV serotypes [82,88] The most common examples are the clinically proven and highly efficient in vivo AAV serotypes 8 and 9, which require a much higher virus load of up to 2-logs of magnitude than other frequently used AAV serotypes to achieve a similar level of encoded gene expression in vitro [89]. 

A number of different approaches were developed to overcome such limitations, including the use of dedicated cell lines for each different AAV serotype in vitro [82,85,90], treating cells with pharmacological drugs to enhance the overall transduction efficiency of gene expression [82,85,90], and co-infection with Adenovirus to boost the replication of AAV genomic copies [85]. However, the protocols described above do not provide options suitable for the entire assortment of available AAV serotypes.

In an effort to resolve the several drawbacks of currently used NAB assays, we recently performed a screening of several drugs that were not previously tested for this purpose and identified a selective inhibitor of AMPK Compound C (CC) as a potent enhancer of HEK293 cell infection by ten different AAV serotypes expressing firefly luciferase. This finding allowed us to develop a serotype-independent, universal NAB assay, which was verified with seropositive serum from mice immunized with individual AAV serotypes [49]. The uniform experimental design of the assay provided us with an opportunity to simultaneously screen patient samples for the presence of NABs against multiple AAV serotypes under identical conditions at the same MOI for each serotype tested. Thus, our assay addressed the previously discussed lack of consistency and standardization in the evaluation of NABs for multiple AAV serotypes [24]. In the current study, we used AAV serotypes 1, 2, 3, 5, 6, 7, 8, 9, 10, and Anc80L65 to analyze the presence of NABs developed during natural AAV infection in plasma samples from dog, NHP, and human donors. 

The two-step strategy we used allowed us to promptly obtain detailed information on AAV-specific seroprevalence for a particular sample. The first step was intended to provide an initial estimation of the presence of NABs against several AAV serotypes, with up to seven in a single assay plate (Figure 2, Figure 4A, and Figure 5A). In the second step, the assay was run against particular AAV serotypes to find the exact titer (Figure 3, Figure 4B, and Figure 5B). In the case of a seronegative sample or NAB titer below the cut-off for clinical study, the second step might be redundant. This strategy is time- and cost-effective and increases the efficiency of large-scale screening efforts. The use of a common commercially available reagent, the HEK 293 cell line, and equipment enables this assay to be set up for nearly any study seeking to further standardize obtained data.

In addition, the development of personalized medicine with the overall intent to treat the patient with a specific therapy and designing appropriate treatment for a patient according to his or her medical information is becoming a widely accepted concept. Personalized, or precision, medicine has shown promising results in some areas of health care, including immune-oncology and cell therapies [91,92,93,94,95]. At the same time, such an approach in gene therapy is limited by the high costs associated with vector production and the small patient population affected by relatively rare genetic diseases. The lack of a personalized approach to find the most appropriate AAV serotype based on personal data precludes a significant number of individuals from receiving the benefits of AAV-based gene therapy. However, new bioprocessing approaches could resolve AAV production bottlenecks [96,97] and will allow off-shelf gene therapy with individually chosen AAV serotypes expressing personalized therapeutic modalities according to patient information. Thus, our NAB assay could be used to identify the AAV serotype(s) that are the most suitable for patients based on their specific NAB profiles (Table 1 and Table 2). However, the ultimate determination of subject eligibility will depend on specific study inclusion criteria, route of administration, target organ or tissues, vector dose, and use of immunosuppressive drugs. 

In summary, in this study, an assay was performed to assess the possibility of evaluating AAV NABs under identical experimental conditions when exactly the same multiplicity of infection was used (2000 vg/cell for each of the ten (10) different AAV serotypes). Validation was performed on a limited (dogs *n* = 6, NHPs *n* = 6, humans *n* = 12) but sufficient number of samples to show the vast difference in NAB profiles for each sample and each AAV serotype tested. Therefore, the universal evaluation of NABs independent of AAV serotype presented in this study further strengthens efforts for the expansion of AAV-based drugs into clinical application.

## Figures and Tables

**Table 1:** Summary of the AAV NAB profile for each individual patient. Blue, yellow, brown, and red correspond to AAV NAB titers ≤2, 2–8, 8–32, and ≥32, respectively.

	AAV1	AAV2	AAV3	AAV5	AAV6	AAV7	AAV8	AAV9	AAV10	AAVANC
H1	<2	<2	<2	<2	<2	<2	<2	<2	<2	<2
H2	<2	2–8	<2	<2	<2	<2	<2	<2	2–8	2–8
H3	<2	2–8	<2	<2	<2	<2	<2	<2	<2	<2
H4	>32	>32	>32	<2	>32	>32	2–8	2–8	>32	>32
H5	8–32	>32	>32	2–8	8–32	<2	8–32	<2	8–32	2–8
H6	<2	2–8	<2	2–8	<2	2–8	2–8	2–8	<2	<2
H7	2–8	8–32	8–32	<2	2–8	8–32	2–8	2–8	8–32	2–8
H8	>32	>32	>32	8–32	>32	>32	8–32	8–32	>32	>32
H9	8–32	>32	>32	<2	2–8	8–32	2–8	2–8	8–32	8–32
H10	2–8	2–8	<2	<2	<2	2–8	2–8	2–8	<2	2–8
H11	8–32	8–32	8–32	2–8	2–8	8–32	2–8	8–32	8–32	2–8
H12	2–8	2–8	2–8	<2	<2	2–8	2–8	2–8	<2	2–8

**Table 2:** Summary of AAV NAB profiles for each individual animal subject. Blue, yellow, brown, and red correspond to AAV NAB titers ≤2, 2–8, 8–32, and ≥32, respectively.

	AAV1	AAV2	AAV3	AAV5	AAV6	AAV7	AAV8	AAV9	AAV10	AAVANC
NHP1	<2	<2	<2	2–8	<2	<2	<2	<2	8–32	<2
NHP2	<2	<2	<2	2–8	<2	<2	<2	2–8	8–32	<2
NHP3	<2	<2	<2	<2	<2	<2	<2	<2	<2	<2
NHP4	<2	<2	<2	8–32	<2	<2	<2	<2	<2	<2
NHP5	<2	2–8	<2	2–8	<2	<2	<2	8–32	2–8	<2
NHP6	2–8	<2	<2	<2	<2	<2	<2	<2	<2	<2
D1	<2	<2	<2	<2	2–8	<2	<2	<2	<2	<2
D2	<2	<2	<2	<2	<2	<2	<2	<2	<2	<2
D3	<2	<2	<2	<2	<2	8–32	<2	2–8	8–32	<2
D4	2–8	<2	<2	<2	<2	<2	<2	<2	<2	<2
D5	8–32	2–8	<2	<2	<2	<2	<2	<2	<2	<2
D6	2–8	>32	2–8	2–8	<2	<2	<2	<2	<2	2–8

## Data Availability

No new data were created or analyzed in this study. Data sharing is not applicable to this article.

## References

[1] Smalley E. (2017). First AAV gene therapy poised for landmark approval. Nat. Biotechnol..

[2] Keeler A.M., Flotte T.R. (2019). Recombinant Adeno-Associated Virus Gene Therapy in Light of Luxturna (and Zolgensma and Glybera): Where Are We, and How Did We Get Here?. Annu. Rev. Virol..

[3] Hacker U.T., Bentler M., Kaniowska D., Morgan M., Buning H. (2020). Towards Clinical Implementation of Adeno-Associated Virus (AAV) Vectors for Cancer Gene Therapy: Current Status and Future Perspectives. Cancers.

[4] Zhan W., Muhuri M., Tai P.W.L., Gao G. (2021). Vectored Immunotherapeutics for Infectious Diseases: Can rAAVs Be The Game Changers for Fighting Transmissible Pathogens?. Front. Immunol..

[5] Mingozzi F. (2018). AAV Immunogenicity: A Matter of Sensitivity. Mol. Ther..

[6] Mingozzi F., High K.A. (2011). Therapeutic in vivo gene transfer for genetic disease using AAV: Progress and challenges. Nat. Rev. Genet..

[7] Calcedo R., Wilson J.M. (2013). Humoral Immune Response to AAV. Front. Immunol..

[8] Halbert C.L., Miller A.D., McNamara S., Emerson J., Gibson R.L., Ramsey B., Aitken M.L. (2006). Prevalence of neutralizing antibodies against adeno-associated virus (AAV) types 2, 5, and 6 in cystic fibrosis and normal populations: Implications for gene therapy using AAV vectors. Hum. Gene Ther..

[9] Li C., Narkbunnam N., Samulski R.J., Asokan A., Hu G., Jacobson L.J., Manco-Johnson M.J., Monahan P.E., Joint Outcome Study I. (2012). Neutralizing antibodies against adeno-associated virus examined prospectively in pediatric patients with hemophilia. Gene Ther..

[10] Boutin S., Monteilhet V., Veron P., Leborgne C., Benveniste O., Montus M.F., Masurier C. (2010). Prevalence of serum IgG and neutralizing factors against adeno-associated virus (AAV) types 1, 2, 5, 6, 8, and 9 in the healthy population: Implications for gene therapy using AAV vectors. Hum. Gene Ther..

[11] Nathwani A.C., Tuddenham E.G., Rangarajan S., Rosales C., McIntosh J., Linch D.C., Chowdary P., Riddell A., Pie A.J., Harrington C. (2011). Adenovirus-associated virus vector-mediated gene transfer in hemophilia B. N. Engl. J. Med..

[12] Verdera H.C., Kuranda K., Mingozzi F. (2020). AAV Vector Immunogenicity in Humans: A Long Journey to Successful Gene Transfer. Mol. Ther..

[13] Louis Jeune V., Joergensen J.A., Hajjar R.J., Weber T. (2013). Pre-existing anti-adeno-associated virus antibodies as a challenge in AAV gene therapy. Hum. Gene Ther. Methods.

[14] Monahan P.E., Negrier C., Tarantino M., Valentino L.A., Mingozzi F. (2021). Emerging Immunogenicity and Genotoxicity Considerations of Adeno-Associated Virus Vector Gene Therapy for Hemophilia. J. Clin. Med..

[15] Wang D., Zhong L., Li M., Li J., Tran K., Ren L., He R., Xie J., Moser R.P., Fraser C. (2018). Adeno-Associated Virus Neutralizing Antibodies in Large Animals and Their Impact on Brain Intraparenchymal Gene Transfer. Mol. Ther. Methods Clin. Dev..

[16] Vandenberghe L.H., Bell P., Maguire A.M., Cearley C.N., Xiao R., Calcedo R., Wang L., Castle M.J., Maguire A.C., Grant R. (2011). Dosage thresholds for AAV2 and AAV8 photoreceptor gene therapy in monkey. Sci. Transl. Med..

[17] Amado D., Mingozzi F., Hui D., Bennicelli J.L., Wei Z., Chen Y., Bote E., Grant R.L., Golden J.A., Narfstrom K. (2010). Safety and efficacy of subretinal readministration of a viral vector in large animals to treat congenital blindness. Sci. Transl. Med..

[18] Boyd R.F., Boye S.L., Conlon T.J., Erger K.E., Sledge D.G., Langohr I.M., Hauswirth W.W., Komaromy A.M., Boye S.E., Petersen-Jones S.M. (2016). Reduced retinal transduction and enhanced transgene-directed immunogenicity with intravitreal delivery of rAAV following posterior vitrectomy in dogs. Gene Ther..

[19] Lee Y.M., Conlon T.J., Specht A., Coleman K.E., Brown L.M., Estrella A.M., Dambska M., Dahlberg K.R., Weinstein D.A. (2018). Long-term safety and efficacy of AAV gene therapy in the canine model of glycogen storage disease type Ia. J. Inherit. Metab. Dis..

[20] Gurda B.L., De Guilhem De Lataillade A., Bell P., Zhu Y., Yu H., Wang P., Bagel J., Vite C.H., Sikora T., Hinderer C. (2016). Evaluation of AAV-mediated Gene Therapy for Central Nervous System Disease in Canine Mucopolysaccharidosis VII. Mol. Ther..

[21] Kruzik A., Fetahagic D., Hartlieb B., Dorn S., Koppensteiner H., Horling F.M., Scheiflinger F., Reipert B.M., de la Rosa M. (2019). Prevalence of Anti-Adeno-Associated Virus Immune Responses in International Cohorts of Healthy Donors. Mol. Ther. Methods Clin. Dev..

[22] Ling C., Wang Y., Feng Y.L., Zhang Y.N., Li J., Hu X.R., Wang L.N., Zhong M.F., Zhai X.F., Zolotukhin I. (2015). Prevalence of neutralizing antibodies against liver-tropic adeno-associated virus serotype vectors in 100 healthy Chinese and its potential relation to body constitutions. J. Integr. Med..

[23] Calcedo R., Vandenberghe L.H., Gao G., Lin J., Wilson J.M. (2009). Worldwide epidemiology of neutralizing antibodies to adeno-associated viruses. J. Infect. Dis..

[24] Weber T. (2021). Anti-AAV Antibodies in AAV Gene Therapy: Current Challenges and Possible Solutions. Front. Immunol..

[25] Perocheau D.P., Cunningham S., Lee J., Antinao Diaz J., Waddington S.N., Gilmour K., Eaglestone S., Lisowski L., Thrasher A.J., Alexander I.E. (2019). Age-Related Seroprevalence of Antibodies Against AAV-LK03 in a UK Population Cohort. Hum. Gene Ther..

[26] Boyce S., James I., Rangarajan S., Curry N., Bagot C., Austin S., Laffan M., Mangles S., Chandrakumaran K., Mundy C. (2022). Seroprevalence to adeno-associated virus type 6 in people with hemophilia B from a UK adult cohort. Res. Pr. Thromb. Haemost..

[27] Daniel H.D., Kumar S., Kannangai R., Lakshmi K.M., Agbandje-Mckenna M., Coleman K., Srivastava A., Srivastava A., Abraham A.M. (2021). Prevalence of Adeno-Associated Virus 3 Capsid Binding and Neutralizing Antibodies in Healthy and Hemophilia B Individuals from India. Hum. Gene Ther..

[28] Nathwani A.C., Reiss U.M., Tuddenham E.G., Rosales C., Chowdary P., McIntosh J., Della Peruta M., Lheriteau E., Patel N., Raj D. (2014). Long-term safety and efficacy of factor IX gene therapy in hemophilia B. N. Engl. J. Med..

[29] Kruzik A., Koppensteiner H., Fetahagic D., Hartlieb B., Dorn S., Romeder-Finger S., Coulibaly S., Weber A., Hoellriegl W., Horling F.M. (2019). Detection of Biologically Relevant Low-Titer Neutralizing Antibodies Against Adeno-Associated Virus Require Sensitive In Vitro Assays. Hum. Gene Ther. Methods.

[30] Ito M., Takino N., Nomura T., Kan A., Muramatsu S.I. (2021). Engineered adeno-associated virus 3 vector with reduced reactivity to serum antibodies. Sci. Rep..

[31] Havlik L.P., Simon K.E., Smith J.K., Klinc K.A., Tse L.V., Oh D.K., Fanous M.M., Meganck R.M., Mietzsch M., Kleinschmidt J. (2020). Coevolution of Adeno-associated Virus Capsid Antigenicity and Tropism through a Structure-Guided Approach. J. Virol..

[32] Li C., Wu S., Albright B., Hirsch M., Li W., Tseng Y.S., Agbandje-McKenna M., McPhee S., Asokan A., Samulski R.J. (2016). Development of Patient-specific AAV Vectors After Neutralizing Antibody Selection for Enhanced Muscle Gene Transfer. Mol. Ther..

[33] Tse L.V., Klinc K.A., Madigan V.J., Castellanos Rivera R.M., Wells L.F., Havlik L.P., Smith J.K., Agbandje-McKenna M., Asokan A. (2017). Structure-guided evolution of antigenically distinct adeno-associated virus variants for immune evasion. Proc. Natl. Acad Sci. USA.

[34] Kotterman M.A., Schaffer D.V. (2014). Engineering adeno-associated viruses for clinical gene therapy. Nat. Rev. Genet..

[35] Pekrun K., De Alencastro G., Luo Q.J., Liu J., Kim Y., Nygaard S., Galivo F., Zhang F., Song R., Tiffany M.R. (2019). Using a barcoded AAV capsid library to select for clinically relevant gene therapy vectors. JCI Insight.

[36] Paulk N.K., Pekrun K., Zhu E., Nygaard S., Li B., Xu J., Chu K., Leborgne C., Dane A.P., Haft A. (2018). Bioengineered AAV Capsids with Combined High Human Liver Transduction In Vivo and Unique Humoral Seroreactivity. Mol. Ther..

[37] Ogden P.J., Kelsic E.D., Sinai S., Church G.M. (2019). Comprehensive AAV capsid fitness landscape reveals a viral gene and enables machine-guided design. Science.

[38] Huttner N.A., Girod A., Perabo L., Edbauer D., Kleinschmidt J.A., Buning H., Hallek M. (2003). Genetic modifications of the adeno-associated virus type 2 capsid reduce the affinity and the neutralizing effects of human serum antibodies. Gene Ther..

[39] Biswas M., Marsic D., Li N., Zou C., Gonzalez-Aseguinolaza G., Zolotukhin I., Kumar S.R.P., Rana J., Butterfield J.S.S., Kondratov O. (2020). Engineering and In Vitro Selection of a Novel AAV3B Variant with High Hepatocyte Tropism and Reduced Seroreactivity. Mol. Ther. Methods Clin. Dev..

[40] Bello A., Tran K., Chand A., Doria M., Allocca M., Hildinger M., Beniac D., Kranendonk C., Auricchio A., Kobinger G.P. (2009). Isolation and evaluation of novel adeno-associated virus sequences from porcine tissues. Gene Ther..

[41] Arbetman A.E., Lochrie M., Zhou S., Wellman J., Scallan C., Doroudchi M.M., Randlev B., Patarroyo-White S., Liu T., Smith P. (2005). Novel caprine adeno-associated virus (AAV) capsid (AAV-Go.1) is closely related to the primate AAV-5 and has unique tropism and neutralization properties. J. Virol..

[42] Farkas S.L., Zadori Z., Benko M., Essbauer S., Harrach B., Tijssen P. (2004). A parvovirus isolated from royal python (*Python regius*) is a member of the genus Dependovirus. J. Gen. Virol..

[43] Elmore Z.C., Oh D.K., Simon K.E., Fanous M.M., Asokan A. (2020). Rescuing AAV gene transfer from neutralizing antibodies with an IgG-degrading enzyme. JCI Insight.

[44] Leborgne C., Barbon E., Alexander J.M., Hanby H., Delignat S., Cohen D.M., Collaud F., Muraleetharan S., Lupo D., Silverberg J. (2020). IgG-cleaving endopeptidase enables in vivo gene therapy in the presence of anti-AAV neutralizing antibodies. Nat. Med..

[45] Chicoine L.G., Montgomery C.L., Bremer W.G., Shontz K.M., Griffin D.A., Heller K.N., Lewis S., Malik V., Grose W.E., Shilling C.J. (2014). Plasmapheresis eliminates the negative impact of AAV antibodies on microdystrophin gene expression following vascular delivery. Mol. Ther..

[46] Salas D., Kwikkers K.L., Zabaleta N., Bazo A., Petry H., van Deventer S.J., Aseguinolaza G.G., Ferreira V. (2019). Immunoadsorption enables successful rAAV5-mediated repeated hepatic gene delivery in nonhuman primates. Blood Adv..

[47] Monteilhet V., Saheb S., Boutin S., Leborgne C., Veron P., Montus M.F., Moullier P., Benveniste O., Masurier C. (2011). A 10 patient case report on the impact of plasmapheresis upon neutralizing factors against adeno-associated virus (AAV) types 1, 2, 6, and 8. Mol. Ther..

[48] Flotte T.R., Trapnell B.C., Humphries M., Carey B., Calcedo R., Rouhani F., Campbell-Thompson M., Yachnis A.T., Sandhaus R.A., McElvaney N.G. (2011). Phase 2 Clinical Trial of a Recombinant Adeno-Associated Viral Vector Expressing α1-Antitrypsin: Interim Results. Hum. Gene Ther..

[49] Krotova K., Aslanidi G. (2020). Modifiers of Adeno-Associated Virus-Mediated Gene Expression in Implication for Serotype-Universal Neutralizing Antibody Assay. Hum. Gene Ther..

[50] Graham M.L., Rieke E.F., Mutch L.A., Zolondek E.K., Faig A.W., Dufour T.A., Munson J.W., Kittredge J.A., Schuurman H.J. (2012). Successful implementation of cooperative handling eliminates the need for restraint in a complex non-human primate disease model. J. Med. Primatol..

[51] Mutch L.A., Klinker S.T., Janecek J.J., Niewinski M.N., Lee R.M.Z., Graham M.L. (2020). Long-Term Management of Vascular Access Ports in Nonhuman Primates Used in Preclinical Efficacy and Tolerability Studies. J. Invest. Surg..

[52] Landegger L.D., Pan B., Askew C., Wassmer S.J., Gluck S.D., Galvin A., Taylor R., Forge A., Stankovic K.M., Holt J.R. (2017). A synthetic AAV vector enables safe and efficient gene transfer to the mammalian inner ear. Nat. Biotechnol..

[53] Pandya J., Ortiz L., Ling C., Rivers A.E., Aslanidi G. (2014). Rationally designed capsid and transgene cassette of AAV6 vectors for dendritic cell-based cancer immunotherapy. Immunol. Cell Biol..

[54] Krotova K., Day A., Aslanidi G. (2019). An Engineered AAV6-Based Vaccine Induces High Cytolytic Anti-Tumor Activity by Directly Targeting DCs and Improves Ag Presentation. Mol. Ther. Oncolytics.

[55] Srivastava A. (2016). Adeno-Associated Virus: The Naturally Occurring Virus Versus the Recombinant Vector. Hum. Gene.

[56] Sabatino D.E., Lange A.M., Altynova E.S., Sarkar R., Zhou S., Merricks E.P., Franck H.G., Nichols T.C., Arruda V.R., Kazazian H.H. (2011). Efficacy and safety of long-term prophylaxis in severe hemophilia A dogs following liver gene therapy using AAV vectors. Mol. Ther..

[57] Sarkar R., Mucci M., Addya S., Tetreault R., Bellinger D.A., Nichols T.C., Kazazian H.H. (2006). Long-term efficacy of adeno-associated virus serotypes 8 and 9 in hemophilia a dogs and mice. Hum. Gene Ther..

[58] Le Guiner C., Montus M., Servais L., Cherel Y., Francois V., Thibaud J.L., Wary C., Matot B., Larcher T., Guigand L. (2014). Forelimb treatment in a large cohort of dystrophic dogs supports delivery of a recombinant AAV for exon skipping in Duchenne patients. Mol. Ther..

[59] Hakim C.H., Yue Y., Shin J.H., Williams R.R., Zhang K., Smith B.F., Duan D. (2014). Systemic gene transfer reveals distinctive muscle transduction profile of tyrosine mutant AAV-1, -6, and -9 in neonatal dogs. Mol. Ther. Methods Clin. Dev..

[60] Yue Y., Shin J.H., Duan D. (2011). Whole body skeletal muscle transduction in neonatal dogs with AAV-9. Methods Mol. Biol..

[61] Stieger K., Colle M.A., Dubreil L., Mendes-Madeira A., Weber M., Le Meur G., Deschamps J.Y., Provost N., Nivard D., Cherel Y. (2008). Subretinal delivery of recombinant AAV serotype 8 vector in dogs results in gene transfer to neurons in the brain. Mol. Ther..

[62] Le Meur G., Stieger K., Smith A.J., Weber M., Deschamps J.Y., Nivard D., Mendes-Madeira A., Provost N., Pereon Y., Cherel Y. (2007). Restoration of vision in RPE65-deficient Briard dogs using an AAV serotype 4 vector that specifically targets the retinal pigmented epithelium. Gene Ther..

[63] Friedman H., Ator N., Haigwood N., Newsome W., Allan J.S., Golos T.G., Kordower J.H., Shade R.E., Goldberg M.E., Bailey M.R. (2017). The Critical Role of Nonhuman Primates in Medical Research. Pathog. Immun..

[64] Jennings M., Prescott M.J., Buchanan-Smith H., Gamble M.R., Gore M., Hawkins P., Hubrecht R., Hudson S., Keeley J.R., Morris K. (2009). Refinements in husbandry, care and common procedures for non-human primates: Ninth report of the BVAAWF/FRAME/RSPCA/UFAW Joint Working Group on Refinement. Lab. Anim..

[65] Flandre T.D., Piaia A., Cary M.G. (2020). Biologic Immunomodulatory Drugs and Infection in the Respiratory Tract of Nonhuman Primates. Toxicol. Pathol..

[66] Heijmans C.M.C., de Groot N.G., Bontrop R.E. (2020). Comparative genetics of the major histocompatibility complex in humans and nonhuman primates. Int. J. Immunogenet..

[67] Estes J.D., Wong S.W., Brenchley J.M. (2018). Nonhuman primate models of human viral infections. Nat. Rev. Immunol..

[68] Miller L.A., Royer C.M., Pinkerton K.E., Schelegle E.S. (2017). Nonhuman Primate Models of Respiratory Disease: Past, Present, and Future. ILAR J..

[69] Garbarini N. (2010). Primates as a model for research. Dis. Model. Mech..

[70] Nathwani A.C., Gray J.T., McIntosh J., Ng C.Y., Zhou J., Spence Y., Cochrane M., Gray E., Tuddenham E.G., Davidoff A.M. (2007). Safe and efficient transduction of the liver after peripheral vein infusion of self-complementary AAV vector results in stable therapeutic expression of human FIX in nonhuman primates. Blood.

[71] Li S., Ling C., Zhong L., Li M., Su Q., He R., Tang Q., Greiner D.L., Shultz L.D., Brehm M.A. (2015). Efficient and Targeted Transduction of Nonhuman Primate Liver With Systemically Delivered Optimized AAV3B Vectors. Mol. Ther..

[72] Guggino W.B., Yanda M.K., Cebotaru C.V., Cebotaru L. (2020). Transduction of Surface and Basal Cells in Rhesus Macaque Lung Following Repeat Dosing with AAV1CFTR. Hum. Gene Ther..

[73] Guggino W.B., Benson J., Seagrave J., Yan Z., Engelhardt J., Gao G., Conlon T.J., Cebotaru L. (2017). A Preclinical Study in Rhesus Macaques for Cystic Fibrosis to Assess Gene Transfer and Transduction by AAV1 and AAV5 with a Dual-Luciferase Reporter System. Hum. Gene Ther. Clin. Dev..

[74] Calcedo R., Franco J., Qin Q., Richardson D.W., Mason J.B., Boyd S., Wilson J.M. (2015). Preexisting Neutralizing Antibodies to Adeno-Associated Virus Capsids in Large Animals Other Than Monkeys May Confound In Vivo Gene Therapy Studies. Hum. Gene Ther. Methods.

[75] Shin J.H., Yue Y., Smith B., Duan D. (2012). Humoral immunity to AAV-6, 8, and 9 in normal and dystrophic dogs. Hum. Gene Ther..

[76] Kuzmin D.A., Shutova M.V., Johnston N.R., Smith O.P., Fedorin V.V., Kukushkin Y.S., van der Loo J.C.M., Johnstone E.C. (2021). The clinical landscape for AAV gene therapies. Nat. Rev. Drug. Discov..

[77] Manno C.S., Pierce G.F., Arruda V.R., Glader B., Ragni M., Rasko J.J., Ozelo M.C., Hoots K., Blatt P., Konkle B. (2006). Successful transduction of liver in hemophilia by AAV-Factor IX and limitations imposed by the host immune response. Nat. Med..

[78] Colella P., Ronzitti G., Mingozzi F. (2018). Emerging Issues in AAV-Mediated In Vivo Gene Therapy. Mol. Ther. Methods Clin. Dev..

[79] Nathwani A.C., Davidoff A.M., Hanawa H., Hu Y., Hoffer F.A., Nikanorov A., Slaughter C., Ng C.Y., Zhou J., Lozier J.N. (2002). Sustained high-level expression of human factor IX (hFIX) after liver-targeted delivery of recombinant adeno-associated virus encoding the hFIX gene in rhesus macaques. Blood.

[80] Guo P., Zhang J., Chrzanowski M., Huang J., Chew H., Firrman J.A., Sang N., Diao Y., Xiao W. (2019). Rapid AAV-Neutralizing Antibody Determination with a Cell-Binding Assay. Mol. Ther. Methods Clin. Dev..

[81] Markusic D.M., Nichols T.C., Merricks E.P., Palaschak B., Zolotukhin I., Marsic D., Zolotukhin S., Srivastava A., Herzog R.W. (2017). Evaluation of engineered AAV capsids for hepatic factor IX gene transfer in murine and canine models. J. Transl. Med..

[82] Falese L., Sandza K., Yates B., Triffault S., Gangar S., Long B., Tsuruda L., Carter B., Vettermann C., Zoog S.J. (2017). Strategy to detect pre-existing immunity to AAV gene therapy. Gene Ther..

[83] Meliani A., Leborgne C., Triffault S., Jeanson-Leh L., Veron P., Mingozzi F. (2015). Determination of anti-adeno-associated virus vector neutralizing antibody titer with an in vitro reporter system. Hum. Gene Ther. Methods.

[84] Mueller C., Tang Q., Gruntman A., Blomenkamp K., Teckman J., Song L., Zamore P.D., Flotte T.R. (2012). Sustained miRNA-mediated knockdown of mutant AAT with simultaneous augmentation of wild-type AAT has minimal effect on global liver miRNA profiles. Mol. Ther..

[85] Wang M., Crosby A., Hastie E., Samulski J.J., McPhee S., Joshua G., Samulski R.J., Li C. (2015). Prediction of adeno-associated virus neutralizing antibody activity for clinical application. Gene Ther..

[86] Fitzpatrick Z., Leborgne C., Barbon E., Masat E., Ronzitti G., van Wittenberghe L., Vignaud A., Collaud F., Charles S., Simon Sola M. (2018). Influence of Pre-existing Anti-capsid Neutralizing and Binding Antibodies on AAV Vector Transduction. Mol. Ther. Methods Clin. Dev..

[87] Food and Drug Administration (FDA) Immunogenicity Testing of Therapeutic Protein Products—Developing and Validating Assays for Anti-Drug Antibody Detection. Guidance for Industry. https://www.regulations.gov/docket?D=FDA-2009-D-0539.

[88] Gorovits B., Fiscella M., Havert M., Koren E., Long B., Milton M., Purushothama S. (2020). Recommendations for the Development of Cell-Based Anti-Viral Vector Neutralizing Antibody Assays. AAPS J..

[89] Gardner M.R., Mendes D.E., Muniz C.P., Martinez-Navio J.M., Fuchs S.P., Gao G., Desrosiers R.C. (2022). High concordance of ELISA and neutralization assays allows for the detection of antibodies to individual AAV serotypes. Mol. Ther. Methods Clin. Dev..

[90] Calcedo R., Chichester J.A., Wilson J.M. (2018). Assessment of Humoral, Innate, and T-Cell Immune Responses to Adeno-Associated Virus Vectors. Hum. Gene Ther. Methods.

[91] Lee J.K., Priceman S.J. (2019). Precision Medicine-Enabled Cancer Immunotherapy. Cancer Treat. Res..

[92] Deng X., Nakamura Y. (2017). Cancer Precision Medicine: From Cancer Screening to Drug Selection and Personalized Immunotherapy. Trends. Pharm. Sci..

[93] Cafri G., Gartner J.J., Zaks T., Hopson K., Levin N., Paria B.C., Parkhurst M.R., Yossef R., Lowery F.J., Jafferji M.S. (2020). mRNA vaccine-induced neoantigen-specific T cell immunity in patients with gastrointestinal cancer. J. Clin. Invest..

[94] Harris E.E.R. (2018). Precision Medicine for Breast Cancer: The Paths to Truly Individualized Diagnosis and Treatment. Int. J. Breast Cancer.

[95] Hamburg M.A., Collins F.S. (2010). The path to personalized medicine. N. Engl. J. Med..

[96] Kotin R.M., Snyder R.O. (2017). Manufacturing Clinical Grade Recombinant Adeno-Associated Virus Using Invertebrate Cell Lines. Hum. Gene Ther..

[97] Adamson-Small L., Potter M., Falk D.J., Cleaver B., Byrne B.J., Clement N. (2016). A scalable method for the production of high-titer and high-quality adeno-associated type 9 vectors using the HSV platform. Mol. Ther. Methods Clin. Dev..

